# Non-diabetic endocrine neuropathies: A practical approach to diagnosis and clinical evaluation

**DOI:** 10.17925/EE.2026.22.2.2

**Published:** 2026-06-24

**Authors:** Marcio J Concepción-Zavaleta, Jenyfer M Fuentes-Mendoza, Luis Concepción-Urteaga, Juan Eduardo Quiroz-Aldave, Arturo Basurto-Ayala, Jose Paz-Ibarra

**Affiliations:** 1. Grupo de Investigación en Neurociencias, Metabolismo, Efectividad Clínica y Sanitaria, Universidad Científica del Sur, Lima, Peru; 2. School of Medicine, Universidad Nacional de Trujillo, Trujillo, Peru; 3. Division of Research, Hospital de Apoyo Chepén, Chepén, Peru; 4. Family Medicine, Hospital Voto Bernales, Lima, Peru; 5. Division of Endocrinology, Hospital Nacional Edgardo Rebagliati Martins School of Medicine, Universidad Nacional Mayor de San Marcos, Lima, Peru

**Keywords:** Acromegaly, algorithm, endocrine system diseases, hypothyroidism, peripheral neuropathy, vitamin B12 deficiency

## Abstract

**Introduction:**

Peripheral neuropathy has diverse etiologies, with endocrine disorders beyond diabetes representing significant, yet frequently underrecognized causes. While diabetic neuropathy is well established, this article specifically excludes a detailed discussion of diabetic neuropathy to focus on less-recognized endocrine causes and presents a practical diagnostic algorithm for their earlier and more accurate identification.

**Methods:**

A focused narrative review was conducted using PubMed, Scopus and ScienceDirect (2015–2025). From 2,318 screened records, 154 studies were included after full-text review of 298 articles. Evidence was synthesized by etiology, mechanism, clinical presentation and diagnostic relevance.

**Results:**

Disorders such as hypothyroidism, acromegaly, adrenal dysfunction and deficiencies of vitamins B12 and D produce distinct neuropathic profiles via metabolic, hormonal, inflammatory or compressive mechanisms. Subclinical nerve involvement is common, with electrophysiological abnormalities detectable in up to 60% of asymptomatic individuals. Characteristic clinical patterns – such as delayed reflexes in hypothyroidism, bilateral carpal tunnel syndrome (CTS) in acromegaly and sensory ataxia in B12 deficiency – provide diagnostic clues. Based on synthesized evidence, a stepwise diagnostic algorithm was developed to guide clinical evaluation.

**Conclusion:**

Endocrine disorders are important and often reversible causes of peripheral neuropathy. Early recognition requires a systematic approach. The proposed evidence-based algorithm offers clinicians a practical tool to improve diagnostic efficiency, facilitate timely treatment of the underlying endocrine condition and reduce the risk of irreversible nerve damage.

Peripheral neuropathy is a common and clinically heterogeneous condition resulting from damage to the peripheral nervous system, affecting sensory, motor and autonomic fibers.^1^ Its diagnostic complexity stems from a broad spectrum of etiologies, including metabolic, autoimmune, infectious, toxic, neoplastic and endocrine disorders.^1,2^

Among endocrine conditions, diabetes mellitus is the most extensively studied and recognized cause of peripheral neuropathy, with well-established diagnostic criteria and management guidelines.^3,4^ However, diabetic neuropathy represents only one component within the broader spectrum of endocrine-related nerve disorders. In contrast, neuropathies associated with other endocrine diseases, such as hypothyroidism, acromegaly, adrenal dysfunction, hypoparathyroidism and vitamin deficiencies, remain comparatively underrecognized despite their potential reversibility.^5–12^

The pathophysiological mechanisms underlying these non-diabetic endocrine neuropathies differ in important ways from classical diabetic sensorimotor polyneuropathy. While chronic hyperglycemia and metabolic dysregulation dominate in diabetes, other endocrine disorders may induce neuropathy through hormonal deficiency or excess, immune-mediated mechanisms, fluid retention and nerve compression, electrolyte imbalance, impaired myelin synthesis or mitochondrial dysfunction.^4,13–17^ These differences are clinically relevant as they may produce distinct neuropathic phenotypes and influence reversibility and therapeutic responses.

Clinically, diabetic neuropathy most commonly presents as distal symmetric polyneuropathy (DSP), characterized by a ‘stocking-glove’ distribution of sensory loss and neuropathic pain.^4,18–20^ In contrast, non-diabetic endocrine neuropathies may manifest with distinctive patterns such as delayed reflex relaxation in hypothyroidism, bilateral CTS in acromegaly, sensory ataxia in vitamin B12 deficiency or neuromuscular hyperexcitability in hypocalcemic states.^21–27^ Although overlap exists between diabetic and non-diabetic neuropathic patterns, early recognition of these distinguishing clinical features is essential for targeted endocrine investigation.

Importantly, several of these non-diabetic endocrine neuropathies are partially or fully reversible when the underlying hormonal disturbance is corrected.^8,20,28,29^ Nevertheless, they are frequently misattributed to more common etiologies such as alcohol-related neuropathy or idiopathic polyneuropathy, particularly when onset is insidious and metabolic screening is incomplete.^2,25^

Given the extensive literature dedicated to diabetic neuropathy, including contemporary international standards of care, this article deliberately centers on endocrine-related peripheral neuropathies beyond diabetes.^3,30^ Our objective is to synthesize current evidence on these less-recognized etiologies, clarify their pathophysiological distinctions and propose a practical, stepwise clinical diagnostic algorithm aimed at improving early identification and reducing preventable nerve damage in routine practice. This article intentionally excludes a detailed discussion of diabetic peripheral neuropathy, which has been extensively addressed elsewhere, and instead focuses on non-diabetic endocrine causes of neuropathy.

## Methods

A structured narrative literature review was conducted to synthesize current evidence on endocrine causes of peripheral neuropathy beyond diabetes, with particular focus on pathophysiological mechanisms, clinical patterns, diagnostic approaches and differential diagnoses.

The literature search was performed using PubMed, Scopus and ScienceDirect databases. The search timeframe was limited to January 2015–July 2025 to capture contemporary evidence, although seminal historical publications were retained when relevant for contextual clarity.

The search strategy combined Medical Subject Headings (MeSH) and free-text keywords including ‘endocrine neuropathy,’ ‘peripheral neuropathy,’ ‘hypothyroidism,’ ‘acromegaly,’ ‘vitamin B12 deficiency,’ ‘adrenal insufficiency,’ ‘growth hormone,’ ‘hypoparathyroidism,’ ‘vitamin D’ and ‘nerve conduction studies.’ Broader terms such as ‘diabetic neuropathy’ were used only to contextualize mechanistic comparisons where necessary.

The initial search yielded 2,318 records. After duplicate removal, 1,655 titles and abstracts were screened for relevance. A full-text review of 298 articles was conducted according to the predefined eligibility criteria.

The inclusion criteria encompassed original research articles, clinical trials, meta-analyses and high-quality reviews published in English that addressed endocrine-related peripheral neuropathy outside the classical diabetic paradigm. Studies exclusively focused on diabetic sensorimotor polyneuropathy without a broader endocrine context were excluded to maintain conceptual clarity and avoid duplication of well-established literature.

Of the excluded full-text articles, a substantial proportion (85 studies) primarily focused on diabetic peripheral neuropathy without a broader endocrine context and were excluded to maintain the specific focus on non-diabetic endocrine etiologies.

From the 298 full-text articles assessed, 152 studies met the final inclusion criteria based on methodological quality and thematic relevance. The reference lists of key articles were manually screened to identify additional pertinent sources.

Data extraction and synthesis followed a thematic framework organized by specific endocrine etiologies, pathophysiological mechanisms, characteristic clinical presentations and diagnostic considerations. Based on this structured evidence synthesis, the diagnostic algorithm presented in this article was developed as a conceptual clinical framework integrating the most consistently reported diagnostic patterns across the literature. The algorithm represents a synthesis of available evidence intended to facilitate clinical reasoning and structured evaluation of endocrine-related neuropathies in routine practice. Future studies should evaluate its clinical utility and diagnostic performance in prospective cohorts.

## Epidemiology

Peripheral neuropathy represents a major global health concern, affecting an estimated 2.5–10% of the general adult population.^31,32^ The burden is substantial, as affected individuals face nearly a fourfold increased risk of lower-limb amputation and reduced overall survival, even after adjustment for comorbidities.^31,33^

Among endocrine conditions, diabetes mellitus remains the most prevalent cause of neuropathy, with up to 50% of patients developing neuropathic involvement over time.^4,34^ Diabetic peripheral neuropathy affects approximately 30% of individuals, with higher prevalence in type two diabetes, and represents a major contributor to global neuropathic disease given the worldwide burden of diabetes.^4,35–38^ In type 1 diabetes, intensive glycemic control reduces the risk and progression of neuropathy, whereas in type 2 diabetes, the benefits of glucose lowering alone are less pronounced, with a greater impact observed from multifactorial risk factor management.^39–42^

Beyond diabetes, a broad spectrum of endocrine disorders – including thyroid dysfunction, adrenal disease, pituitary disorders and metabolic deficiencies – have been associated with peripheral nerve involvement, although their true epidemiological burden remains less clearly defined.^43^

However, neuropathies associated with non-diabetic endocrine disorders are epidemiologically less well characterized, partly due to underdiagnosis and heterogeneous clinical presentation. In overt hypothyroidism, peripheral neuropathy has been reported in up to 20–40% of patients, often presenting subclinically.^44,45^ In acromegaly, neurological involvement – including entrapment neuropathies and polyneuropathy – has been documented in up to 50–80% of cases, depending on disease activity and diagnostic method.^22,46^ Hypercortisolism is frequently associated with proximal myopathy, while overt peripheral neuropathy appears less common but remains underreported.^47,48^ Vitamin B12 deficiency-related neuropathy is particularly relevant in older populations and individuals with malabsorption, with prevalence estimates ranging between 10 and 15% in at-risk groups.^49^

Electrophysiological studies further suggest that subclinical nerve conduction abnormalities may be present in 40–60% of patients with chronic endocrine disorders, even in the absence of overt neuropathic symptoms.^50^ These findings underscore the importance of systematic neurological evaluation in patients with sustained endocrine dysfunction, particularly when symptoms are subtle or nonspecific.

Overall, while diabetes accounts for the largest proportion of endocrine-related neuropathies globally, non-diabetic endocrine etiologies represent a clinically meaningful and potentially reversible subset that warrants greater epidemiological and diagnostic attention.

## Metabolic and hormonal contributions to nerve injury

Although not the focus of this article, hyperglycemia is briefly included as a reference mechanism given its well-established role in peripheral neuropathy, allowing comparison with less-recognized endocrine neuropathies that constitute the primary focus of this study.

### Hypothyroidism

Thyroid hormone deficiency significantly impacts peripheral nerve function through multiple mechanisms. Hypothyroidism disrupts myelin synthesis and axonal transport, with experimental models demonstrating reduced myelinated fiber density and delayed axonal diameter increase in sciatic nerves.^45,51^ Schwann cell proliferation and remyelination capacity are substantially impaired in thyroid-deficient states.^44,45^ Thyroid hormones are essential for developmental myelination, critically regulating the maturation of both oligodendrocyte and Schwann cell lineages.^52^

Beyond direct effects on myelin, hypothyroidism promotes peripheral nerve damage through fluid retention mechanisms, causing tissue edema that can lead to nerve compression. In advanced cases such as myxedema, impaired slow axonal transport contributes to axonal degeneration and clinical neuropathy.^45^ These findings underscore the essential role of thyroid hormones in maintaining myelin integrity and supporting normal axonal function.

### Cortisol excess or deficiency

Dysregulation of cortisol homeostasis, encompassing both hypercortisolism (Cushing’s syndrome) and hypocortisolism (adrenal insufficiency), contributes to peripheral nerve injury through neuroinflammatory mechanisms and altered neuronal excitability. Clinical evidence demonstrates that elevated serum cortisol independently predicts diabetic neuropathy risk, with patients in the highest cortisol quartiles showing nearly double the risk compared to those in the lowest quartiles, even after adjusting for glycemic control and other metabolic parameters.^53^

Hypothalamic–pituitary–adrenal axis dysregulation appears central to this process, with altered cortisol dynamics correlating with neuropathic pain severity in various conditions, including chemotherapy-induced neuropathy.^54^ Excessive cortisol may disrupt white matter microstructure, promoting nerve demyelination, as evidenced by neuroimaging studies in patients with Cushing’s syndrome.^55^ Glucocorticoid excess potentiates neuroinflammatory responses through receptor-mediated microglial activation and pro-inflammatory cytokine release, while cortisol deficiency impairs neuronal energy metabolism and disrupts neuroimmune homeostasis, compromising peripheral nerve repair mechanisms.^53^

### Excess growth hormone/insulin-like growth factor-1

In acromegaly, excess growth hormone (GH) and insulin-like growth factor-1 (IGF-1) promote neuropathy primarily through mechanical compression and tissue edema. Median nerve edema, demonstrable by increased cross-sectional area on magnetic resonance imaging (MRI) and ultrasonography, underlies the high prevalence of CTS in this population.^56,57^ The generalized edema characteristic of active acromegaly results from GH-enhanced epithelial sodium channel activity in the distal nephron, promoting sodium reabsorption and fluid retention that affects nerve tissues.^57^

Paradoxically, while IGF-1 exhibits neurotrophic and regenerative properties under physiological conditions, promoting Schwann cell support and axonal elongation, its chronic excess in acromegaly contributes to maladaptive tissue overgrowth that increases nerve compression risk. This dysregulated IGF-1 signaling may simultaneously impair endogenous repair mechanisms while exacerbating compressive neuropathy.^58^

### Vitamin B12 deficiency

Vitamin B_₁₂_ deficiency induces peripheral nerve injury through two key cobalamin-dependent reactions that are critical for myelin integrity and neuronal bioenergetics. First, adenosylcobalamin serves as a cofactor for methylmalonyl-CoA mutase, which converts methylmalonyl-CoA to succinyl-CoA. In B_₁₂_ deficiency, an impaired activity of this enzyme leads to accumulation of methylmalonyl-CoA and methylmalonic acid (MMA). These metabolites are thought to disrupt normal lipid metabolism and promote incorporation of abnormal fatty acids into myelin, ultimately impairing myelin stability and favoring demyelination. This MMA-related pathway is classically implicated in the demyelinating process underlying subacute combined degeneration (SCD).^59,60^

Second, methylcobalamin is required for methionine synthase, which converts homocysteine to methionine and supports the formation of S-adenosylmethionine, a universal methyl donor essential for methylation reactions involved in myelin maintenance. A dysfunction of this pathway contributes to impaired myelin synthesis and neuronal repair and may act synergistically with MMA-mediated lipid dysregulation to produce combined central and peripheral nervous system injury.^59^

Clinically, B12 deficiency may manifest as mixed neuroaxis involvement, combining peripheral neuropathy with myelopathy, most characteristically SCD of the spinal cord, which affects the dorsal columns and corticospinal tracts and may present with sensory ataxia, loss of vibration/proprioception, spasticity and upper motor neuron signs. As neurological manifestations can occur even when hematologic abnormalities are subtle or absent, functional assessment using MMA (and/or homocysteine) can improve diagnostic sensitivity in suspected cases.^59–61^

### Vitamin D deficiency

Vitamin D exerts neuroprotective effects through calcium homeostasis regulation, oxidative stress reduction and modulation of neurotrophic and inflammatory pathways. Deficiency states are associated with increased peripheral neuropathy risk and severity, particularly in people with diabetes^62^ Observational studies consistently demonstrate that patients with diabetic neuropathy have lower serum 25(OH)D levels compared with those without neuropathy, with deficiency correlating with slower nerve conduction velocities and higher neuropathic pain scores.^3^

Intervention studies suggest a potential therapeutic role for vitamin D supplementation in vitamin D-deficient patients with diabetic neuropathy, particularly for short-term reduction in neuropathic pain; some trials also report improvement in inflammatory markers such as interleukin-6, although evidence for tumor necrosis factor-α reduction and for consistent improvement in nerve conduction parameters remains limited (*Figure 1*).^63,64^

**Figure 1:**
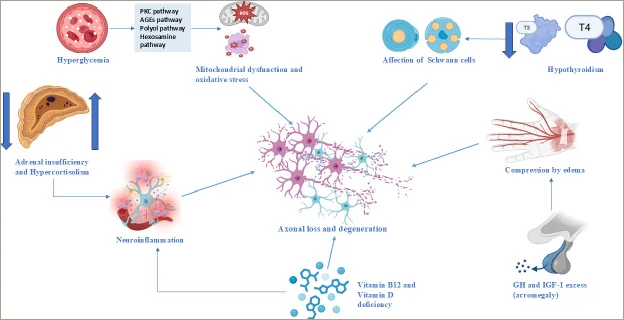
Metabolic and hormonal contributions to nerve injury

*AGE = advanced glycation end-products; GH = growth hormone; IGF-1 = insulin-like growth factor 1; PKC = protein kinase C; T3 = triiodothyronine; T4 = thyroxine.*

## Major endocrine causes of neuropathy

Diabetes mellitus is the most common endocrine cause of peripheral neuropathy.^65^ Its clinical spectrum includes distal symmetric, autonomic and small fiber neuropathies, driven by mechanisms such as chronic hyperglycemia, oxidative stress, inflammation and microvascular injury.^13^ Additionally, prediabetes and insulin resistance are associated with early nerve damage, often manifesting as small fiber and autonomic dysfunction prior to overt diabetes.^66–68^

The following sections focus on the spectrum of non-diabetic endocrine disorders associated with peripheral neuropathy, highlighting their distinct pathophysiological mechanisms and clinical presentations.

The following sections focus on the spectrum of non-diabetic endocrine disorders associated with peripheral neuropathy, highlighting their distinct pathophysiological mechanisms and clinical presentations.

### Hypothyroidism

Hypothyroidism induces neurological manifestations through multiple pathways. The characteristic-delayed relaxation phase of deep tendon reflexes (Woltman’s sign), particularly affecting the Achilles reflex, demonstrates a 72% positive predictive value for diagnosis.^21^ This phenomenon results from thyroid hormone deficiency, reducing muscle ATPase activity and impairing calcium reuptake in the sarcoplasmic reticulum, leading to slowed muscle contraction-relaxation cycles and subclinical sensorimotor neuropathy.^21,69^ Additionally, mucopolysaccharide accumulation within the carpal tunnel causes median nerve compression, mimicking CTS (*Table 1*).^70^

### Acromegaly

Acromegaly frequently presents with neurological complications, with CTS affecting 25–64% of patients as an early manifestation.^22,71^ Soft tissue and synovial hypertrophy driven by excess GH compresses the median nerve, producing characteristic sensory symptoms. Comprehensive clinical assessments reveal neurological involvement in 87.5% of patients with acromegaly, with CTS present in 50% and polyneuropathy in 29.2%, the latter being more prevalent in cases with uncontrolled biochemical disease (*Table 1*).^46^

### Cushing’s syndrome and Addison’s disease

Excess glucocorticoid in Cushing’s syndrome commonly produces proximal myopathy, affecting 40–70% of patients, particularly in lower limbs.^26^ The pathophysiology involves activation of the FOXO signaling pathway, which upregulates the muscle-specific E3 ubiquitin ligases atrogin-1 (MAFbx) and MuRF-1, promoting protein degradation through ubiquitin–proteasome and autophagy systems. These mechanisms preferentially affect fast-twitch type 2 fibers, leading to muscle atrophy, intramuscular fat accumulation and persistent weakness.^72–74^ Rare disorders such as adrenoleukodystrophy and adrenomyeloneuropathy combine adrenal insufficiency with demyelinating neuropathy through ABCD1 gene mutations that impair very long-chain fatty acid metabolism, leading to toxic lipid accumulation that damages both central and peripheral nervous systems along with adrenal cortex function (*Table 1*).^75,76^

### Vitamin B12 deficiency

Vitamin B12 deficiency causes SCD, characterized by dorsal column and lateral corticospinal tract damage.^49^ Clinical presentation includes progressive paresthesias, weakness, sensory ataxia, impaired vibration and proprioception, spasticity, Babinski signs and occasionally cognitive changes. MRI typically demonstrates T2 hyperintensity in the posterior spinal cord, often displaying an ‘inverted V’ configuration or longitudinal involvement.^26^ Pernicious anemia represents the most common etiology, featuring autoimmune attack against intrinsic factor or gastric parietal cells, frequently associated with chronic autoimmune gastritis, gastric hyperplasia and neuroendocrine tumors.^49^ Medication-induced deficiency from metformin, proton pump inhibitors and H2 receptor antagonists also contributes significantly to B12 malabsorption (*Table 1*).^49^

### Hypoparathyroidism

Parathyroid hormone deficiency leads to hypocalcemia, which increases neuromuscular excitability by lowering the threshold for neuronal depolarization through effects on voltage-gated sodium channels. Clinically, this manifests as muscle cramps, tetany and the classic Chvostek and Trousseau signs.^77^ Chronic hypocalcemia has also been associated with sensory-motor peripheral neuropathy, a manifestation that may improve following correction of calcium levels with calcium and vitamin D supplementation (*Table 1*).^78^

**Table 1:** Major endocrine disorders associated with peripheral neuropathy: clinical manifestations, mechanisms and prevalence

Endocrine cause	Mechanism	Manifestations	Clinical points	Alterations	Estimated prevalence
Diabetes mellitus	Chronic hyperglycemia, oxidative stress and mitochondrial dysfunction	Distal symmetric polyneuropathy, autonomic dysfunction and small fiber neuropathy	Occurs in up to 50% of diabetics after 10 years; tight glucose control helps	↓ nerve conduction velocity, axonal degeneration and microangiopathy	Up to 50% of diabetics
Prediabetes and insulin resistance	Insulin resistance and metabolic dysfunction	Small fiber neuropathy and autonomic symptoms	Can precede diabetes; nerve damage before hyperglycemia is evident	↓ epidermal nerve fiber density and autonomic testing abnormalities	20–40% of insulin-resistant individuals
Hypothyroidism	Thyroid hormone deficiency affecting neuronal ATPase activity	Paresthesia, hypoesthesia, delayed reflexes and mimics carpal tunnel	Symptoms may be subclinical; often seen in chronic cases	↓ sensory and motor nerve conduction and entrapment neuropathies	20% in overt hypothyroidism
Acromegaly	GH-induced soft tissue and nerve compression	Sensory deficits, carpal tunnel syndrome and peripheral neuropathy	87.5% of patients show neurological involvement	Nerve compression signs, EMG changes in CTS	Up to 87.5% of acromegaly cases
Cushing’s syndrome and Addison’s disease	Glucocorticoid excess (FOXO3 activation) and rare VLCFA accumulation	Muscle weakness, atrophy, demyelinating neuropathy in rare cases	Seen in 40–70% of Cushing’s; Addison’s has less data, ABCD1 linked to adrenoleukodystrophy	EMG: myopathy; VLCFA: demyelination on MRI/EEG	40–70% in Cushing’s syndrome
Vitamin B12 deficiency	B12-dependent myelin synthesis disruption	Paresthesia, ataxia, cognitive dysfunction and weakness	Reversible with early treatment; long-term cases may have lasting damage	MRI: spinal cord involvement; EMG: mixed neuropathy	10–15% of elderly or malnourished patients
Hypoparathyroidism and hypocalcemia	Low PTH and hypocalcemia causing neuromuscular instability	Tetany, muscle cramps and positive Chvostek/Trousseau signs	Rapidly reversible with calcium and vitamin D supplementation	EMG: hyperexcitability; ECG changes if systemic	Rare; common in postsurgical or autoimmune cases
Vitamin D deficiency	Calcium/phosphate imbalance affecting nerve function and NGF	Muscle weakness, allodynia and neuropathic pain	Often overlooked; may worsen other deficiencies and cause chronic pain	↓ NGF levels, ↑ pain sensitivity and abnormal QST findings	Very common but underdiagnosed

*ABCD1 = ATP-binding cassette subfamily D member 1; ATPase = adenosine triphosphatase; CTS = carpal tunnel syndrome; ECG = electrocardiography; EEG = electroencephalography; EMG = electromyography; FOXO3 = Forkhead Box O3; GH = growth hormone; MRI = magnetic resonance imaging; NGF = nerve growth factor; PTH = parathyroid hormone; QST = quantitative sensory testing; VLCFA = very long-chain fatty acids.*

### Vitamin D deficiency

Vitamin D deficiency has been increasingly associated with neuromuscular symptoms and neuropathic pain beyond glycemic contexts. Through the activation of the vitamin D receptor in neural and glial tissues, vitamin D may influence peripheral nerve homeostasis via immunomodulatory, neurotrophic and myelin-related pathways.^79,80^ Experimental models demonstrate that cholecalciferol (vitamin D_₃_) promotes axogenesis, enhances myelin gene expression and supports structural and functional nerve recovery, partly through Schwann cell-mediated mechanisms.^80,81^

Deficiency has also been linked to increased neuroinflammatory signaling and altered nociceptive processing, potentially contributing to peripheral hypersensitivity and neuropathic pain.^79,82,83^ Clinically, vitamin D deficiency should be considered a potentially reversible contributor to neuropathic symptoms, particularly when other causes are absent, although high-quality trials are still needed to clarify therapeutic benefit (*Table 1*).^79,80^

## Clinical manifestations

Endocrine-related peripheral neuropathies commonly present as a distal, symmetric sensory (± sensorimotor) syndrome with numbness, paresthesias, burning pain and reduced vibration/ankle reflexes, reflecting a length-dependent process that can overlap with the classic diabetic DSP phenotype.^30^ Beyond diabetes, similar bilateral presentations may occur in metabolic/endocrine states such as prediabetes/insulin resistance, thyroid dysfunction and vitamin deficiencies, where small fiber involvement can predominate (pain, allodynia and temperature dysesthesia) even when routine nerve conduction studies (NCS) are normal.^43,84^ In vitamin B12 deficiency, symptoms may extend beyond peripheral neuropathy to include myelopathic features – sensory ataxia, loss of proprioception/vibration, spasticity – consistent with SCD, which should be suspected when ‘peripheral neuropathy + posterior column signs’ coexist.^60^

Several endocrinopathies have a strong association with entrapment neuropathies, particularly median nerve compression. In acromegaly, CTS is frequent and may be bilateral; it can precede the endocrine diagnosis and should prompt consideration of excess GH/IGF-1 when accompanied by suggestive systemic features.^6^ In hypothyroidism, tissue edema and mucopolysaccharide deposition can contribute to compressive neuropathies (including CTS) and may coexist with mild symmetric polyneuropathy.^84^ Overall, separating presentations into symmetric length-dependent neuropathy versus focal/asymmetric entrapment patterns helps guide targeted endocrine testing and avoids over-attribution to diabetes alone.^43^

## Clinical approach

### Clinical suspicion and initial assessment

A high index of suspicion for endocrine-related neuropathy should be maintained in patients with known endocrine disorders or those presenting with neuropathic symptoms. Up to 50% of individuals with prediabetes demonstrate detectable nerve damage through clinical or electrophysiological testing, even before meeting the formal diabetes diagnostic criteria.^85^ Early manifestations of vitamin deficiencies may be subtle, presenting as intermittent paresthesias, lower extremity heaviness or distal sensory disturbances.^86^

In early-stage hypothyroidism, small fiber dysfunction – characterized by burning pain, dysesthesia, allodynia and autonomic symptoms – may occur despite normal NCS and electromyography (EMG). Recent studies have demonstrated somatosensory and autonomic alterations, reporting a reduction in intraepidermal nerve fibre density (IENFD) as a potential biomarker for preclinical small fiber neuropathy in patients with hypothyroidism.^87,88^

Systematic screening is, therefore, recommended for all patients with prediabetes, established diabetes, thyroid dysfunction or known nutritional deficiencies, regardless of symptom presence, utilizing validated clinical algorithms from initial evaluation.^65^

### Neuropathic pain screening questionnaires

In patients presenting with chronic pain suggestive of peripheral neuropathy, validated screening questionnaires can support an early identification of a neuropathic pain component prior to confirmatory testing. According to recent evidence and guidelines, tools such as the Douleur Neuropathique en 4 Questions (DN4), Leeds Assessment of Neuropathic Symptoms and Signs (LANSS) and painDETECT have been formally recommended for use in the diagnostic workup of possible neuropathic pain, with DN4 and LANSS receiving the strongest endorsement.^89^ These instruments assess characteristic sensory descriptors (e.g. burning and electric shock-like pain) and symptom distribution patterns, helping to stratify symptoms and increase diagnostic confidence. While they do not replace neurological examination or electrophysiological testing, their structured approach is supported in contemporary recommendations for early recognition and diagnostic guidance.^89,90^

### Comprehensive physical examination

A structured neurological examination forms the cornerstone of neuropathy assessment. Sensory evaluation should encompass multiple modalities, including light touch, vibration perception and pinprick testing, as their combined assessment provides high sensitivity for early peripheral nerve dysfunction detection.^91^ Deep tendon reflex assessment, particularly evaluation of the Achilles reflex, offers valuable diagnostic information as it is frequently impaired in diabetic neuropathies.^92^ The examination should also include gait analysis and motor strength testing to identify distal weakness, foot drop or wrist/finger abnormalities, with attention to muscle atrophy or deformities that may indicate advanced motor involvement.^51^

### Phenotype triage

For a chronic, length-dependent DSP phenotype, a limited high-yield work-up is appropriate. Atypical features (acute/subacute onset, marked asymmetry, predominant motor deficits or prominent autonomic failure) warrant expedited neurology referral and expanded testing.^93^

### Targeted laboratory investigation

Laboratory assessment should be guided by clinical findings and suspected underlying endocrine pathophysiology. In diabetes, both chronic hyperglycemia (reflected by hemoglobin A1c levels) and glycemic variability correlate with neuropathy severity and progression.^94^ Thyroid function testing is essential as even subclinical hypothyroidism demonstrates genetic association with peripheral neuropathy risk.^84^ Calcium and parathyroid hormone evaluation should be considered given the neuromuscular manifestations of calcium-phosphate imbalances.^88^ For vitamin B12 status assessment, MMA and homocysteine measurements provide enhanced sensitivity over serum B12 alone, identifying functional deficiencies that may contribute to progressive nerve injury despite apparently normal B12 levels.^95^

In patients presenting with a typical DSP, initial high-yield screening should also include evaluation for paraproteinemia (serum protein electrophoresis with immunofixation), alongside glucose testing and vitamin B12 with metabolites.^96^

### Electrophysiological characterization

NCS and EMG provide critical objective data for neuropathy characterization and differential diagnosis. In diabetic neuropathy, NCS typically demonstrates slowed nerve conduction velocities, reduced sensory and motor amplitudes, prolonged distal latencies and F-wave abnormalities, reflecting mixed axonal and demyelinating pathology.^97^ Conversely, neuropathies associated with hypothyroidism, parathyroid disorders or significant vitamin B12 deficiency typically exhibit predominantly axonal patterns, characterized by reduced amplitudes on NCS and denervation changes on EMG (*Figure 2*).^98^

If symptoms strongly suggest neuropathic pain/small fiber involvement but NCS/EMG are normal, consider small fiber assessment (e.g. QST, skin biopsy for IENFD and/or corneal confocal microscopy where available).^99^

## Differential diagnosis

The evaluation of peripheral neuropathy necessitates a broad differential diagnosis, as numerous conditions beyond endocrine disorders can present with similar symptoms. A systematic approach is essential to distinguish endocrine-related neuropathies from other common etiologies, which include toxic, nutritional, autoimmune, paraneoplastic and infectious causes. The key distinguishing features of these alternative diagnoses are outlined below.

### Alcoholic neuropathy

Alcoholic neuropathy represents one of the most frequent neurological complications of chronic alcohol use disorder, affecting up to 66% of this patient population.^100,101^ The pathogenesis involves both direct neurotoxic effects of ethanol and its metabolites and malnutrition-induced nerve damage. Women and individuals with positive family history appear at higher risk for developing more severe and rapidly progressive polyneuropathy.^102^ Chronic consumption exceeding 100 g/day significantly increases neuropathy risk, typically manifesting as painful paresthesias and sensory ataxia with predominant distal lower extremity involvement.^103^ Management centers on alcohol cessation and nutritional repletion, particularly with thiamine, vitamin B12 and folate supplementation.^104^

### Nutritional deficiencies

Nutritional neuropathies may present as isolated vitamin deficiencies or complex nutritional syndromes. Thiamine deficiency (beriberi) manifests with cardiovascular involvement, fatigue, irritability and distal sensory-motor symptoms including burning pain and muscle weakness. Notably, approximately 25% of patients with thiamine-deficient polyneuropathy concurrently develop Wernicke’s encephalopathy, necessitating prompt parenteral thiamine replacement at initial doses of 100 mg daily.^105^ Niacin deficiency (pellagra), characterized by the classic triad of dermatitis, diarrhea and dementia, results from inadequate dietary intake of fish, meats and legumes, with treatment focusing on nicotinamide supplementation.^106,107^

**Figure 2:**
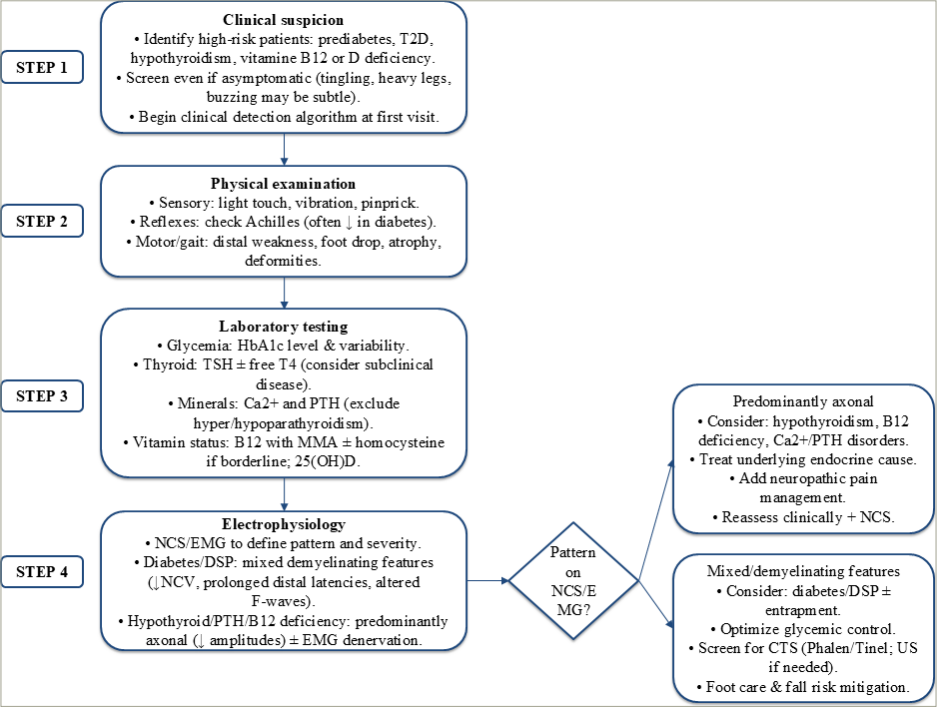
Clinical algorithm for the evaluation of endocrine-related peripheral neuropathy

*25(OH)D = 25-hydroxyvitamin D; CTS = carpal tunnel syndrome; EMG = electromyography; DSP = distal symmetric polyneuropathy; HbA1c = hemoglobin A1c; MMA = methylmalonic acid; NCS = nerve conduction studies; NCV = nerve conduction velocity; PTH = parathyroid hormone; T2D = type 2 diabetes; TSH = thyroid-stimulating hormone; US = ultrasound.*

### Autoimmune neuropathies

Chronic inflammatory demyelinating polyradiculoneuropathy (CIDP) represents an immune-mediated disorder characterized by progressive, symmetric weakness affecting both distal and proximal muscles, accompanied by sensory disturbances and diminished tendon reflexes developing over at least 8 weeks.^108,109^ CIDP frequently coexists with various systemic conditions, including diabetes, Sjögren’s syndrome, hepatitis, human immunodeficiency virus (HIV) and thyroid disorders.^108^ Vasculitic neuropathies, whether isolated to the peripheral nervous system or part of systemic vasculitis, involve inflammatory destruction of vasa nervorum, leading to ischemic nerve damage and typically presenting as mononeuritis multiplex.^110,111^

### Paraneoplastic syndromes

Paraneoplastic neurological syndromes occur as remote effects of malignancy, most commonly associated with lung cancer, gynecological tumors and lymphomas.^112^ Paraneoplastic polyneuropathies rank among the most frequent neurological complications of systemic cancer, often characterized by acute or subacute onset and cerebrospinal fluid lymphocytic pleocytosis, sometimes revealing previously undiagnosed malignancy.^113,114^

### Infectious causes

Infectious agents can induce peripheral neuropathy through direct microbial damage or immune-mediated mechanisms.^115^ HIV represents the most extensively characterized viral cause, with DSP affecting 30–50% of patients with advanced disease, typically presenting with stocking-glove distribution sensory symptoms.^116,117^ Hepatitis C virus infection may produce neurological manifestations, including arthralgias, palpable purpura and peripheral neuropathy.^118^ Lyme neuroborreliosis typically manifests within the first 2 months of infection, causing radiculopathies, cranial neuropathies, mononeuropathies or diffuse polyneuropathies, requiring prompt recognition and appropriate antimicrobial therapy.^119^

### Chemotherapy-induced peripheral neuropathy

Chemotherapy-induced peripheral neuropathy (CIPN) is a common, dose-limiting neurotoxic complication of many anticancer agents (e.g. platinum compounds and taxanes), occurring in ~30–70% of treated patients. It primarily affects sensory fibers, leading to numbness, tingling, burning pain and altered temperature or vibration perception in a glove-and-stocking distribution; motor and autonomic symptoms can also occur. CIPN may persist long after treatment cessation and significantly impacts quality of life and functional status.^120,121^

## Management

Recent European Academy of Neurology (EAN)-European Pain Federation (EFIC)-Neuropathic Pain Special Interest Group (NeuPSIG) recommendations emphasize evidence-based approaches for diagnosing and characterizing neuropathic pain (e.g. validated questionnaires, skin biopsy and quantitative sensory testing) and align management with phenotype and mechanism rather than etiology alone. They focus on diagnostic rigor and tailored assessment tools.^122^

In contrast, the National Institute for Health and Care Excellence (NICE) guideline provides practical pharmacotherapy guidance for neuropathic pain in non-specialist settings, offering specific drug recommendations and safety considerations (e.g. duloxetine, gabapentin and lidocaine/capsaicin topical agents) but placing less emphasis on detailed phenotypic stratification.^123^

### Addressing underlying endocrine dysfunction

Metabolic control plays a role in endocrine-related neuropathies; however, its impact varies depending on the underlying endocrine disorder and is more established in diabetes mellitus than in other conditions.^40,41^ In type one diabetes, intensive glycemic control reduces neuropathy risk and progression, whereas in type 2 diabetes, benefits are less direct and depend on multifactorial management.^40,41,124^ Lifestyle interventions may improve function but rarely reverse established neuropathy.^125^

Adjunctive pharmacological approaches, particularly angiotensin-converting enzyme inhibitors such as quinapril, demonstrate additional neuroprotective benefits through modulation of the renin–angiotensin system.^126^ For hypothyroidism, levothyroxine replacement (typically initiated when TSH exceeds 10 mIU/L) frequently alleviates neuropathic symptoms, while cortisol normalization remains essential in managing Cushing’s syndrome-associated neuropathy.^28,127,128^

### Neuropathic pain management

Neuropathic pain management in endocrine-related neuropathies follows general principles, although most available evidence derives from studies in diabetic neuropathy.

Neuropathic pain management requires a stratified approach.^129^ First-line agents include duloxetine (a serotonin–norepinephrine reuptake inhibitor) and pregabalin, both US Food and Drug Administration-approved with meta-analyses confirming their benefits for pain control and quality of life.^130–132^ Gabapentin, though primarily an anticonvulsant, is also recommended for various neuropathic pain conditions, including postherpetic neuralgia.^133,134^ Second-line options such as tramadol or conventional opioids are reserved for refractory cases due to concerns regarding dependence and adverse effects, while cannabinoids have emerged as third-line therapies for specific conditions including HIV-associated neuropathy (*Table 2*).^118–123,125–127,134–137^

### Adjunctive therapies

Complementary treatments play a supportive role in neuropathy management. Neurotropic B vitamins (B1, B6 and B12) act synergistically to promote nerve regeneration through antioxidant mechanisms and myelin maintenance.^29,138,139^ Alpha-lipoic acid demonstrates efficacy in reducing oxidative stress associated with diabetic neuropathy.^140^ Vitamin D supplementation shows potential for symptom improvement in deficient patients, though its precise therapeutic role requires further elucidation.^79^

### Multidisciplinary care

Comprehensive management necessitates a multidisciplinary approach incorporating neurology, endocrinology and physical therapy expertise to optimize functional recovery in patients with polyneuropathies. While this collaborative model represents optimal care, its implementation raises important questions regarding cost-effectiveness and long-term sustainability that warrant further investigation.^141^

## Prognosis and long-term outcomes

Prognosis in endocrine-related neuropathies critically depends on early diagnosis and sustained management of the underlying endocrine disorder. Although stringent glycemic control in diabetes may slow neurological deterioration, established nerve damage often proves irreversible, with 28–50% of patients developing chronic symptoms or complications such as foot ulcers.^4,34,36^ Neuropathies associated with hypothyroidism or vitamin B12 deficiency typically show improvement with appropriate hormone or vitamin replacement, though delayed intervention may result in incomplete recovery.^28,29^ For compressive neuropathies, such as those occurring in acromegaly, symptom resolution closely correlates with biochemical disease control.^57,85^ The frequent detection of subclinical nerve abnormalities through electrophysiological testing in asymptomatic at-risk patients underscores the importance of routine screening for early intervention.^96^

## Future directions

Emerging technologies promise transformative advances in diagnosing and managing endocrine-related neuropathies. Biomarker discovery has identified methylglyoxal and superoxide dismutase activity as significant contributors to diabetic peripheral neuropathy, while long non-coding RNAs (e.g. NONRATT021972) correlate with neuropathic pain intensity and represent potential therapeutic targets.^142,143^ Non-invasive techniques such as corneal confocal microscopy and skin biopsies quantifying intraepidermal nerve fiber density enable objective assessment of small fiber damage, even during subclinical stages.^144–147^ Artificial intelligence applications, including tools like INSPIRE, are revolutionizing diagnostic precision by integrating raw waveform analysis with comprehensive clinical data.^148,149^ Furthermore, personalized risk prediction models that combine biomarkers (e.g. neurofilament light chain and fibroblast growth factor-19) with demographic and clinical variables show promising accuracy for identifying diabetic sensorimotor polyneuropathy, potentially reducing dependence on costly diagnostic procedures.^150,151^ These innovations collectively signal a paradigm shift toward precision medicine in neuropathy care.

**Table 2:** Dosing regimens, adverse effects and recommendations for currently neuropathic pain drugs^118–123,125–127^

Current neuropathic pain drugs						
	Drug	Initial dose	Dose range	Adverse effects	Recommendations	References
First-line analgesics	Gabapentin	100–300 mg/d	300–1200 mg three times/d	Drowsiness, dizziness, peripheral edema, visual blurring and weight gain	Dosage adjustments required in renal failure and in elderly patients	^122,123^
	Pregabalin	25–150 mg/d	150–300 mg twice daily	Drowsiness, dizziness, peripheral edema, visual blurring and weight gain	Dosage adjustments required in renal failure	^119,121^
	Duloxetine	30 mg/d	60–120 mg/d	Sedation, nausea, constipation, ataxia, dry mouth and loss of appetite	Contraindicated in patients with glaucoma and blood pressure follow-up	^119,121^
	Venlafaxine	37.5 mg/d	150–225 mg/d	Nausea, dizziness, drowsiness, hyperhidrosis and hypertension	Dosage adjustments required in renal failure and blood pressure follow-up	^118,120^
Second-line analgesics	Tramadol	50 mg/d	50–100 mg four times/d or 100–400 mg/d (controlled release)	Ataxia, sedation, constipation, seizures and orthostatic hypertension	Associated with seizures in patients at high seizure risk or when combined with medications that increase serotonin level	^123,125^
	5% lidocaine patches	NA	Apply to painful areas for 12 h in a 24 h period	NA	Most useful for postherpetic neuralgia	^123,126^
Third-line analgesics	Cannabinoids	Varies by product	Individualized titration	Somnolence, confusion, dizziness, tachycardia and hypotension	Not appropriate in those who are younger than 25 years of age, pregnant, cardiovascular disease, respiratory disease, history of psychosis or a substance use disorder. Consider only in specific cases (e.g. HIV neuropathy) and assess risk of dependence	^127^

*HIV = human immunodeficiency virus; NA = not available.*

## Keypoints for clinical practice

- Systematically screen for neuropathy in all patients with prediabetes, diabetes, thyroid disorders or nutritional deficiencies, as up to 50% may have detectable nerve damage before symptom onset.
- Recognize distinctive neuropathic patterns that suggest specific endocrine etiologies: delayed reflexes in hypothyroidism, bilateral carpal tunnel in acromegaly and sensory ataxia in vitamin B12 deficiency.
- Implement targeted laboratory testing beyond standard neuropathy workup, including TSH, vitamin B12 with methylmalonic acid, HbA1c and cortisol when clinically indicated.
- Prioritize correction of the underlying endocrine dysfunction as the primary therapeutic strategy, as many endocrine-related neuropathies are potentially reversible with timely intervention.
- Adopt a tiered approach to neuropathic pain management, beginning with first-line agents (duloxetine and pregabalin) while reserving opioids for refractory cases due to dependency risks.
- Engage multidisciplinary care early, combining endocrinology for metabolic control, neurology for diagnostic precision and physical therapy for functional recovery.

## Conclusions

The evidence demonstrates that endocrine-related peripheral neuropathies remain consistently underdiagnosed despite showing distinct clinical patterns and measurable biochemical and electrophysiological abnormalities. Early identification through structured clinical assessment, targeted endocrine testing and NCS is essential, as many of these neuropathies are at least partially reversible when the underlying hormonal disturbance is corrected. The stepwise diagnostic algorithm proposed in this article represents an evidence-informed synthesis of current literature designed to support clinical reasoning during the evaluation of endocrine-related neuropathies. It provides a practical framework for structured assessment and may help clinicians recognize potentially reversible endocrine causes earlier in the diagnostic process.
